# Subtyping and risk model construction based on taurine metabolism-related genes for predicting prognosis and immune response in lung adenocarcinoma

**DOI:** 10.1515/med-2026-1389

**Published:** 2026-07-20

**Authors:** Lei Wu, Minzhe Li, Jia Zhang

**Affiliations:** Department of Respiratory and Critical Care Medicine, The First Affiliated Hospital of Air Force Medical University, Xi’an, Shaanxi, China; Department of Respiratory and Critical Care Medicine, Lequn Branch of the First Hospital of Jilin University, Changchun, Jilin, China; Department of Respiratory and Critical Care Medicine, The Affiliated Hospital of Yanbian University (Yanbian Hospital), Yanji, Jilin, China

**Keywords:** lung adenocarcinoma, taurine metabolism, prognostic model, subtypes, immune response

## Abstract

**Objectives:**

Lung adenocarcinoma (LUAD) remains a major cause of cancer-related deaths worldwide. While taurine has been shown to suppress tumor growth in laboratory models, its relationship with LUAD patient survival remains unclear. This study constructs a taurine metabolism-based prognostic model for LUAD to predict survival and immunotherapy response, supporting personalized treatment.

**Methods:**

Using transcriptomic and clinical data from public databases (TCGA and GEO), we identified prognostic genes linked to taurine metabolism in LUAD. Patients were classified into two molecular subtypes using clustering analysis. Key genes were verified experimentally (qRT-PCR), and a 9-gene risk model was developed using machine learning approaches. We then analyzed immune infiltration, tumor mutations, immunotherapy response, and drug sensitivity across subtypes and risk groups. Regulatory networks involving transcription factors and miRNAs were also constructed.

**Results:**

A 9-gene signature effectively stratified LUAD patients into high- and low-risk groups. High-risk patients showed activated tumor growth pathways and an immune-suppressive microenvironment, resulting in poorer survival and limited response to immunotherapy. In contrast, low-risk patients exhibited favorable outcomes and enhanced immune activity. We also identified STAT3/GATA2 and hsa-miR-98-5p as key regulatory factors.

**Conclusions:**

This study highlights the important role of taurine metabolism in LUAD progression and immunity. The proposed gene signature offers a new tool for prognosis prediction and may help guide personalized treatment strategies.

## Introduction

Lung cancer is a major contributor to global cancer incidence and mortality, with a generally poor prognosis [1]. As the most common subtype of non-small cell lung cancer, lung adenocarcinoma (LUAD) accounts for approximately 40 % of all lung cancer cases [2]. It typically originates in the peripheral glands of the lung and shows a relatively higher incidence among women and non-smokers [3]. Breakthroughs in immunotherapy for LUAD, particularly the use of PD-1/PD-L1 inhibitors as representative ICIs, have provided new treatment avenues [4]. Particularly for patients with high tumor mutational burden (TMB) or high PD-L1 expression, immunotherapy may yield favorable outcomes [5], 6]. In 2020, a comprehensive proteomic analysis of 103 Chinese LUAD patients revealed molecular characteristics associated with the disease, their relationship to clinical prognosis, and identified potential prognostic biomarkers and druggable targets [7]. Another study demonstrated that Ect2 not only contributes to *in vivo* tumor formation but also possesses a novel ribosome-related function in LUAD cells [8]. Furthermore, with deepening research into the LUAD tumor microenvironment (TME), scientists are exploring novel therapeutic strategies that modulate the TME to enhance treatment efficacy.

Taurine, a sulfur-containing amino acid, represents the most abundant free amino acid in humans and performs diverse biological functions [9]. It demonstrates potent antioxidant properties by scavenging free radicals and reactive oxygen species (ROS), thereby contributing to the maintenance of mitochondrial function [10]. It offers multiple benefits for the cardiovascular system, including blood pressure regulation and anti-inflammatory functions, contributing to a reduced risk of cardiovascular diseases [11]. In recent years, growing attention has been focused on the role of taurine in cancer biology. Taurine exerts pro-apoptotic and anti-proliferative effects across cancer cell lines. In lung cancer models, it upregulates PUMA and Bax while downregulating Bcl-2. It inhibits cell proliferation in a concentration- and time-dependent manner and significantly increases the apoptosis rate [12]. It suppresses LUAD by modulating tumor microenvironment, including regulating T/B cell proliferation and antagonizing M1 macrophage polarization [13], 14]. Taurine also plays a significant role in tumor metabolic reprogramming and immune regulation. In bladder cancer, tumor cells overexpress SLC6A6 to excessively uptake taurine, causing CD8^+^ T cells to develop endoplasmic reticulum stress due to taurine deficiency, which activates the ATF4 signaling pathway, upregulates immune checkpoint molecules including PD-1 and TIM-3, and ultimately induces an immunosuppressive microenvironment and anti-PD-1 therapy resistance [15], 16]. Additionally, taurine and proline synergistically activate the aryl hydrocarbon receptor, inducing a Treg-macrophage immunosuppressive axis that forms an immune escape microenvironment [17]. The association between taurine metabolism and LUAD prognosis remains elusive, though its elucidation could facilitate novel metabolism-based cancer therapies.

However, the association between taurine metabolism and LUAD prognosis remains elusive, and the potential of these metabolic regulators as clinical biomarkers has not been fully explored. We hypothesized that the dysregulation of taurine metabolism-related genes (TMRGs) remodels the tumor immune microenvironment, thereby driving LUAD progression and influencing therapeutic efficacy. Consequently, this study aims to address the following research question: Can a robust genomic signature derived from TMRGs effectively stratify LUAD patients by prognosis and accurately predict their response to immunotherapy and chemotherapy? To answer this, we integrated multi-omics analysis to construct a risk model and validated its predictive value across multiple cohorts.

## Materials and methods

### Data collection

RNA-seq data for LUAD (526 tumor and 59 normal samples) were obtained from the UCSC Xena database (https://xena.ucsc.edu/; accessed June 3, 2025). The latest TCGA LUAD copy number variation data and matching clinical records (https://portal.gdc.cancer.gov/; accessed June 3; 2025) were also downloaded, excluding patients without survival information. Validation set transcriptomic data for LUAD (GSE30219, GSE31210, GSE42127, GSE72094) originated from the GEO database (https://www.ncbi.nlm.nih.gov/geo/; accessed June 4, 2025). A total of 610 TMRGs were identified from existing literature [9].

### Characterization of genes associated with taurine metabolism

Differential gene expression between LUAD tumor and normal tissues was performed using edgeR (v4.2.2). Genes meeting |logFC|>1 and FDR<0.05 were defined as differentially expressed genes (DEGs). Differentially expressed taurine metabolism-related genes (DETMRGs) were identified by intersecting the DEGs with the pre-defined set of taurine metabolism-related genes. After excluding patients with survival <30 days, prognosis-associated DETMRGs were identified by univariate Cox regression (p<0.05). These genes’ expression was compared across normal and tumor groups, followed by Pearson correlation analysis to evaluate inter-gene relationships. Finally, genes were analyzed in the STRING database (v12.0, interaction score >0.4, https://cn.string-db.org/; accessed June 4, 2025) to construct a protein-protein interaction (PPI) network, visualized with Cytoscape v3.10.2.

### Identification of taurine metabolism-related subtypes

LUAD samples were classified into two subtypes (k=2) using Non-negative Matrix Factorization (NMF) based on the expression profile of prognosis-related DETMRGs. For the NMF method, the standard “brunet” option was selected and 30 iterations were carried out. The number of clusters k was set as 2–10, the average contour width of the common membe matrix was determined through the NMF package. The cophenetic, dispersion and silhouette indicators were used to determine the optimal clustering number, the optimal clustering number selected was two. Survival analysis was subsequently performed on the identified subgroups using the R survival package (v3.8-3). For validation, tumor samples from four GEO datasets (GSE30219, GSE31210, GSE42127, and GSE72094) were subtyped using the same DETMRGs, partitioned into identical subgroups, and subsequently analyzed for survival outcomes with the same package. Boxplots were generated to visualize the expression patterns of prognosis-related DETMRGs across the different subtypes. Furthermore, differential gene expression analysis between subtypes was conducted (|logFC|>1, FDR<0.05), followed by GO enrichment analysis of DEGs.

### Screening prognostic-related features to construct prognostic model

A prognostic model was constructed using 41 prognosis-related DETMRGs. To mitigate overfitting, LASSO regression analysis was performed on the candidate prognostic genes using glmnet package (v4.1-10), with the optimal penalty parameter (lambda) selected via cross-validation to eliminate highly correlated genes and reduce model complexity. LASSO regression selected the optimal penalty parameter (lambda) through 10-fold cross-validation. The lambda value (lambda.min) corresponding to the minimum cross-validation error was taken as the optimal lambda. Multivariate Cox regression applied to LASSO-selected genes established a refined prognostic model. To further optimize variable selection in the model, the stepwise regression was performed using the step function with a bidirectional approach, allowing up to 5,000 iterations. The Akaike Information Criterion (AIC) was employed as the model selection criterion to iteratively add or remove variables, ultimately identifying a set of genes with independent prognostic value. Each patient’s risk score was computed from signature gene expression weighted by their regression coefficients. Using the median risk score as cutoff, patients were stratified into high- and low-risk groups. Survival curves and ROC curves (via timeROC package (v0.4)) were generated, with area under the curve (AUC) values calculated for 1-, 3-, and 5-year survival. Calibration curves were used to evaluate the accuracy of model predictions. A composite plot depicted survival status and risk distribution across both groups. External validation was performed using four GEO datasets (GSE30219, GSE31210, GSE42127, and GSE72094), with corresponding ROC curves, survival curves, and combined survival status-risk score distribution plots generated for each dataset. Additionally, survival analysis was conducted for individual signature genes, and Kaplan-Meier curves were plotted using the TCGA training cohort to evaluate their prognostic significance.

### GSEA and pathway enrichment assessment

Based on the prognostic model, samples were assigned risk scores and stratified into high- and low-risk groups using the median risk score as the cutoff. GSEA v4.3.2 executed pathway enrichment for both groups. Concurrently, DEGs between risk groups were identified. These DEGs underwent GO/KEGG enrichment analysis using clusterProfiler package (v4.12.6).

### Independent prognostic evaluation and clinical subgroup stratification

To assess the prognostic model’s icndependence, univariate and multivariate Cox analyses incorporating clinical characteristics and risk scores were conducted. A nomogram predicting 1-, 3-, and 5-year survival was built using the “rms” R package (v8.0-0), with calibration curves validating its accuracy. Decision curve analysis (DCA) evaluated clinical utility. Violin plots were generated to examine the association between risk scores and clinical features (age ≤65 vs. >65, gender, TNM stage), while Kaplan–Meier survival analysis was conducted to quantify survival differences across clinical subgroups.

### Immune analysis and immunotherapy response prediction

ssGSEA (GSVA package (v1.52.3)) uses a gene set of 29 immune cell types or functions from Supplementary Table 1 as background, with parameters set to method=“ssgsea” and kcdf=“Gaussian,” quantifying immune infiltration scores for cell types/functions in subtypes and risk groups. The “estimate” R package (v1.0.13) computed Immune/Stromal/ESTIMATE Scores and Tumor Purity for these cohorts. CIBERSORT (v1.03) used the LM22 signature matrix and ran 1,000 permutations (perm=1,000) to analyze immune infiltration differences. Immune checkpoint gene expression was compared across groups. IPS scores (TCIA: https://tcia.at; accessed June 6, 2025) and TIDE scores (http://tide.dfci.harvard.edu/; accessed June 6, 2025) for TCGA-LUAD were obtained, with comparative analyses of high-/low-risk groups. The IMvigor210 cohort [18] assessed anti-PD-L1 response.

### Tumor mutational burden profiling

Mutation profiles from TCGA-LUAD cohorts were analyzed to calculate TMB scores for individual samples. Mutation patterns of the top 20 mutated genes in each risk group were analyzed and visualized via the GenVisR package (v1.36.0).

### Pharmacogenomic sensitivity assessment

Candidate drugs demonstrating significant correlations between sensitivity profiles and signature gene expression were identified through the CellMiner database (https://discover.nci.nih.gov/cellminer/; accessed March 27, 2024) (Supplementary Table 2). The pRRophetic package (v0.5) predicted therapeutic compounds’ IC50 values in high- and low-risk subgroups.

### Prediction of potential TF and construction miRNA-target gene regulatory networks

Potential miRNAs targeting signature genes were identified using the miRNet database (v2.0, https://www.mirnet.ca/; accessed July 1, 2025), while upstream transcription factors (TFs) were predicted via NetworkAnalyst (v3.0, https://www.networkanalyst.ca/; accessed July 1, 2025). All networks were visualized in Cytoscape v3.10.2.

### Cell acquisition and cell culture

Human lung epithelial cells (BEAS-2B), human lung cancer cells (A549) and human lung cancer cells (HCC827) cell lines were obtained from the American Type Culture Collection (ATCC) and cultured in DMEM medium. All cell cultures were maintained in a 37 °C incubator with 5 % CO_2_. In the cytotoxicity assay, A549 was the high-risk group cell line, while HCC827 was the low-risk group cell line.

### Quantitative reverse transcription polymerase chain reaction (qRT-PCR)

Total RNA was extracted from tissues or cultured cell lines (BEAS-2B and A549) using the Total RNA Kit (Yeasen, Shanghai, China), following the manufacturer’s protocol. Complementary DNA (cDNA) was synthesized from 1 μg of total RNA using the Hifiar III 1st Strand cDNA Synthesis SuperMix for qPCR (with genomic DNA digestion) and the cDNA Synthesis Kit (Yeasen). For quantitative PCR (qPCR), Hieff UNICON Universal Blue qPCR SYBR Master Mix (Yeasen) was used. Data were normalized to the expression of the reference gene, β-actin, to control for variations in expression levels. All experiments were independently repeated three times. The sequences of the primers used are listed in Table 1.

**Table 1:** Sequences of primers for qRT-PCR.

Gene name	Primer sequences (5′–3′)
PRKCZ	Forward: GTT​CTC​CTG​GTG​CGG​TTG​AAG​A
	Reverse: GGT​TGC​TGG​ATG​CCT​GCT​CAA​A
MS4A14	Forward: GAC​CTC​ATA​GCT​CTC​TGC​TGG​A
	Reverse: GGA​AGT​CTT​TGG​GAG​AAA​CCG​AT
F12	Forward: CTC​TGT​CCA​CAA​CAC​CTC​ACT​G
	Reverse: ATC​AGG​ACC​CTT​GCA​CTG​GCA​T
PDX1	Forward: GAA​GTC​TAC​CAA​AGC​TCA​CGC​G
	Reverse: GGA​ACT​CCT​TCT​CCA​GCT​CTA​G
ABCC8	Forward: GAC​GAC​AAG​AGG​ACA​GTG​GTC​T
	Reverse: GCA​TTC​AGA​CCT​CTG​GAA​GTC​C
BDNF	Forward: CAT​CCG​AGG​ACA​AGG​TGG​CTT​G
	Reverse: GCC​GAA​CTT​TCT​GGT​CCT​CAT​C
TIMP1	Forward: GGA​GAG​TGT​CTG​CGG​ATA​CTT​C
	Reverse: GCA​GGT​AGT​GAT​GTG​CAA​GAG​TC
CFTR	Forward: GGA​GAG​CAT​ACC​AGC​AGT​GAC​T
	Reverse: TTC​CAA​GGA​GCC​ACA​GCA​CAA​C
FMO3	Forward: TGG​AAA​GCG​TGT​CCT​GGT​GGT​T
	Reverse: TCA​TCA​CCC​AGG​AGC​CAC​TTC​T
β-actin	Forward: CAC​CAT​TGG​CAA​TGA​GCG​GTT​C
	Reverse: AGG​TCT​TTG​CGG​ATG​TCC​ACG​T

### Cytotoxicity assay

Using the 3-(4, 5-dimethylthiazol-2-yl)-2, 5-diphenyltetrazolium bromide (MTT) method to determine the toxic effects of drugs on cells A549 and HCC827 cells were seeded into a 96 well plate. After cell attachment, the cells were treated with various concentrations of Cisplatin or Docetaxel for 48 h, including a blank control group. Subsequently, 10 μL of MTT solution was added to each well and the plate was incubated at 37 °C for 2 h. The supernatant was then aspirated, and DMSO was added to dissolve the formazan. The absorbance of each well was measured at a wavelength of 490 nm.

### Statistical analysis

Statistical analyses used R software (v4.4.1). Wilcoxon tests assessed inter-group differences; Pearson/Spearman correlations evaluated variable associations. Significance threshold: p<0.05.

## Results

### Analysis of DETMRGs in LUAD patients

**Figure 1:**
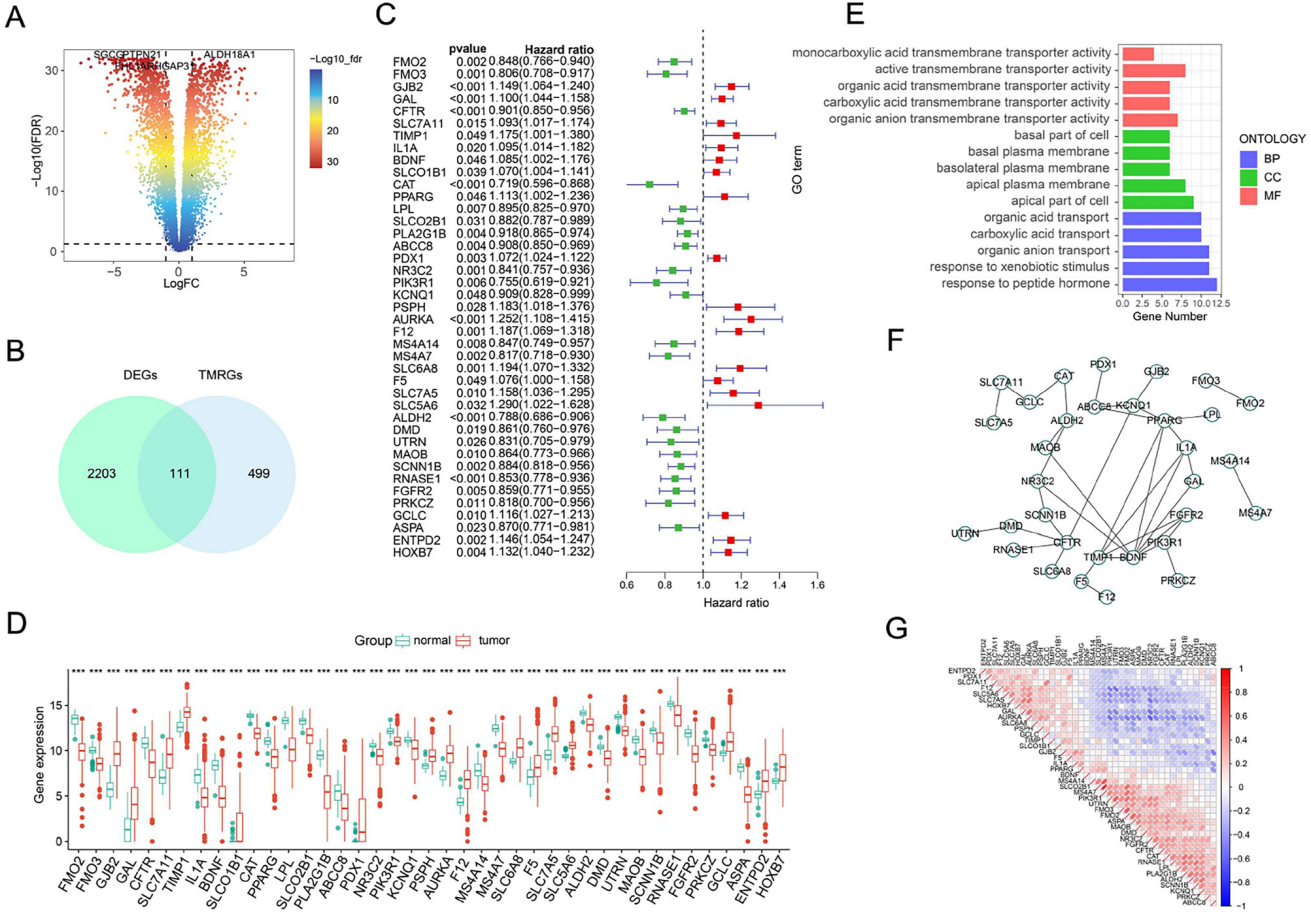
DETMRGs identification and functional analysis. (A) DEG volcano plot (FDR<0.05, |log_2_FC|>1) (B) DEGs and taurine metabolism genes Venn diagram. (C) Forest plot of 41 prognosis-associated DETMRGs (univariate Cox, p<0.01). (D) Boxplot of expression levels for 41 genes in tumor vs. normal groups. (E) GO enrichment analysis of 41 prognostic genes. (F) PPI network analysis of prognostic DETMRGs. (G) correlation heatmap of prognostic DETMRGs. ns: not significant, *p<0.05, **p<0.01, ***p<0.001.

Integration of TCGA-LUAD datasets, differential gene analysis identified 2,314 DEGs (FDR<0.05, |logFC|>1), including 1,040 upregulated and 1,274 downregulated genes (Figure 1A). By intersecting DEGs with taurine metabolism-related genes, we identified 111 DETMRGs (Figure 1B). Univariate Cox regression (p<0.05) further identified 41 prognostically relevant genes (Figure 1C, Supplementary Table 3). Expression analysis of these 41 genes between tumor and normal groups revealed significant differential expression patterns (Figure 1D). The GO Functional enrichment analysis of these 41 prognostic genes revealed significant enrichment in pathways related to monocarboxylic acid transport activity, various cellular membrane components, and the transport of organic and carboxylic acids (Figure 1E). PPI network analysis and heatmap visualization further elucidated functional relationships and co-expression patterns among these genes (Figure 1F and G).

### Subtype identification and associated analyses

**Figure 2:**
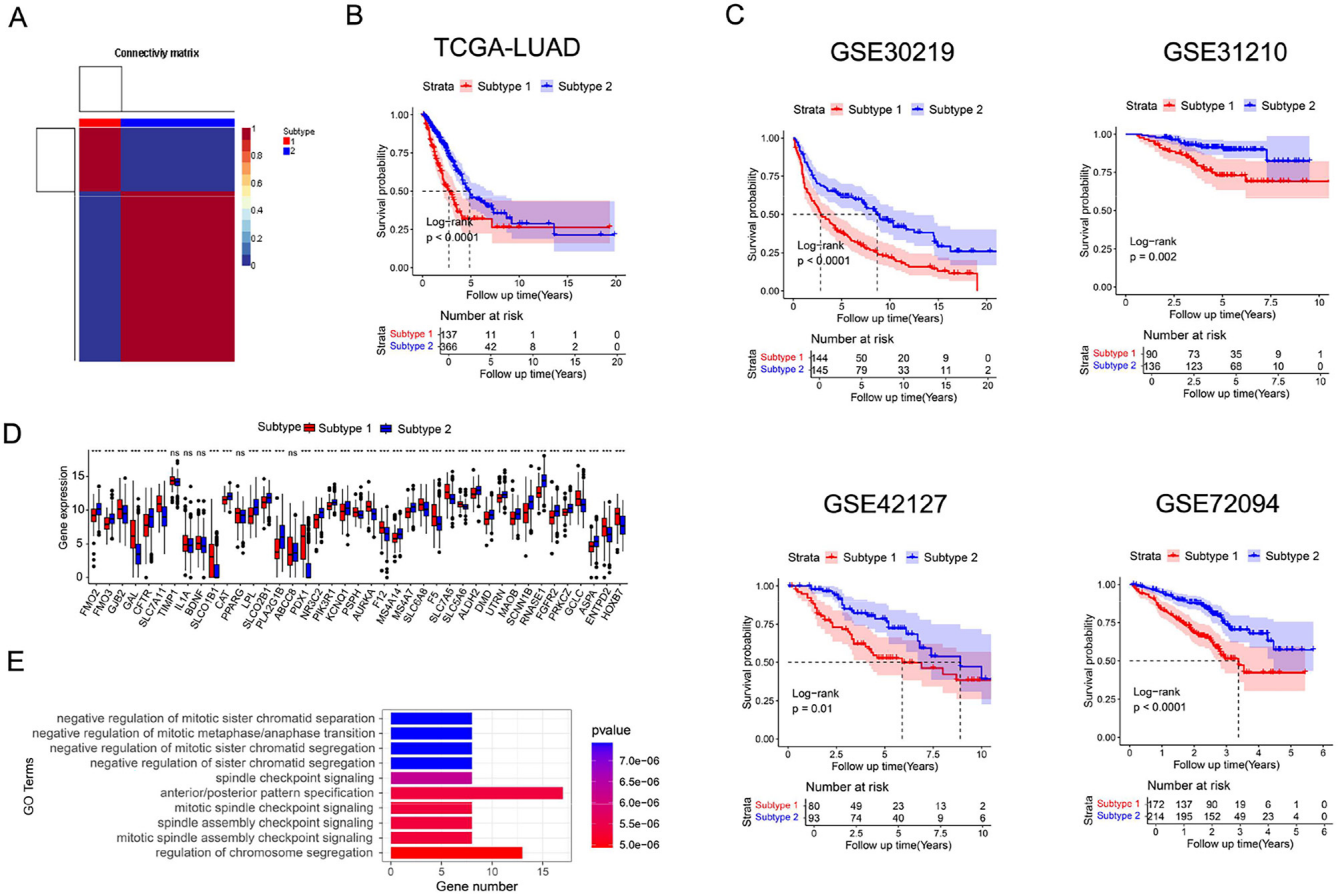
Subtype identification and survival analysis. (A) NMF clustering stratified the 41 prognostic genes into two distinct subtypes. (B) Cluster-based survival analysis in TCGA-LUAD. (C) Cluster-based survival analysis in validation datasets (GSE30219, GSE31210, GSE42127, and GSE72094). (D) 41-gene expression boxplots in subtypes. (E) inter-subtype DEGs GO enrichment. ns: not significant, *p<0.05, **p<0.01, ***p<0.001.

Based on 41 prognostic-related DETMRGs, NMF clustering analysis was performed to stratify LUAD patients. The optimal cluster number K=2 was determined by evaluating multiple metrics including cophenetic correlation coefficient and silhouette score results (Supplementary Figure 1). The clustering matrix at K=2 clearly divided into two distinct blocks (Figure 2A). Survival analysis revealed that patients in subtype1 had a significantly lower survival rate (Figure 2B). The tumor samples were also successfully classified into two subtypes across four GEO validation datasets (GSE30219, GSE31210, GSE42127, and GSE72094), with subtype1 consistently exhibiting a lower survival rate (Figure 2C). Expression analysis of the 41 prognostic genes between the two subtypes revealed significant differential expression for most genes (Figure 2D). Functional enrichment analysis of DEGs between subtypes identified significant involvement in biological processes related to chromosome segregation (Figure 2E).

### Immune profiling and clinical characterization of subtypes

**Figure 3:**
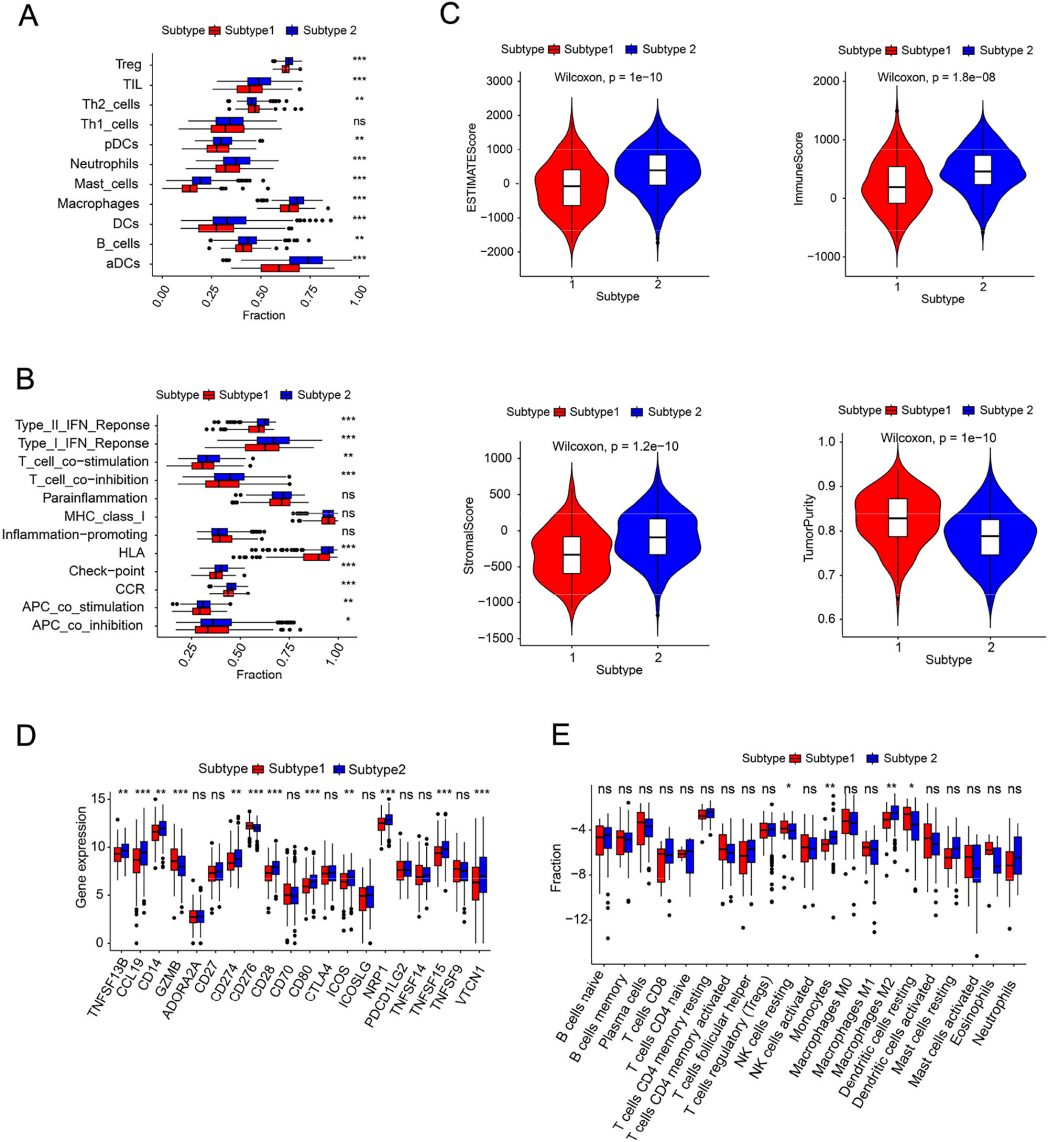
Immune microenvironment characterization. (A) ssGSEA immune cell and (B) immune function scores boxplot for different subtype groups. (C) ESTIMATE analysis of tumor microenvironment components. (D) Differential immune checkpoint expression between different subtype groups. (E) CIBERSORT immune cell fractions in different subtype groups. ns: not significant, *p<0.05, **p<0.01, ***p<0.001.

Analysis using the ssGSEA algorithm revealed that Subtype 1 exhibited significantly lower levels of multiple immune cell populations compared to Subtype 2, including B cells, Mast cells, Macrophages, and Neutrophils, along with reduced immune function scores (Figure 3A and B). ESTIMATE analysis showed subtype 1 with significantly lower Immune/Stromal/ESTIMATE Scores, while subtype 2 had reduced Tumor Purity (Figure 3C). Immune checkpoint genes differed markedly between subtypes (Figure 3D). Subtype 1 exhibited significantly elevated expression of GZMB and CD276, whereas Subtype 2 showed markedly higher expression levels of TNFSF13B, CCL19, CD14, CD274, CD28, CD80, ICOS, NRP1, TNFSF15, and VTCN1. CIBERSORT analysis further revealed that Subtype 1 exhibited significantly increased infiltration of NK cells resting and Dendritic cells resting, whereas Subtype 2 showed elevated infiltration of Macrophages M2 and monocytes (Figure 3E).

### Prognostic model construction and assessment

**Figure 4:**
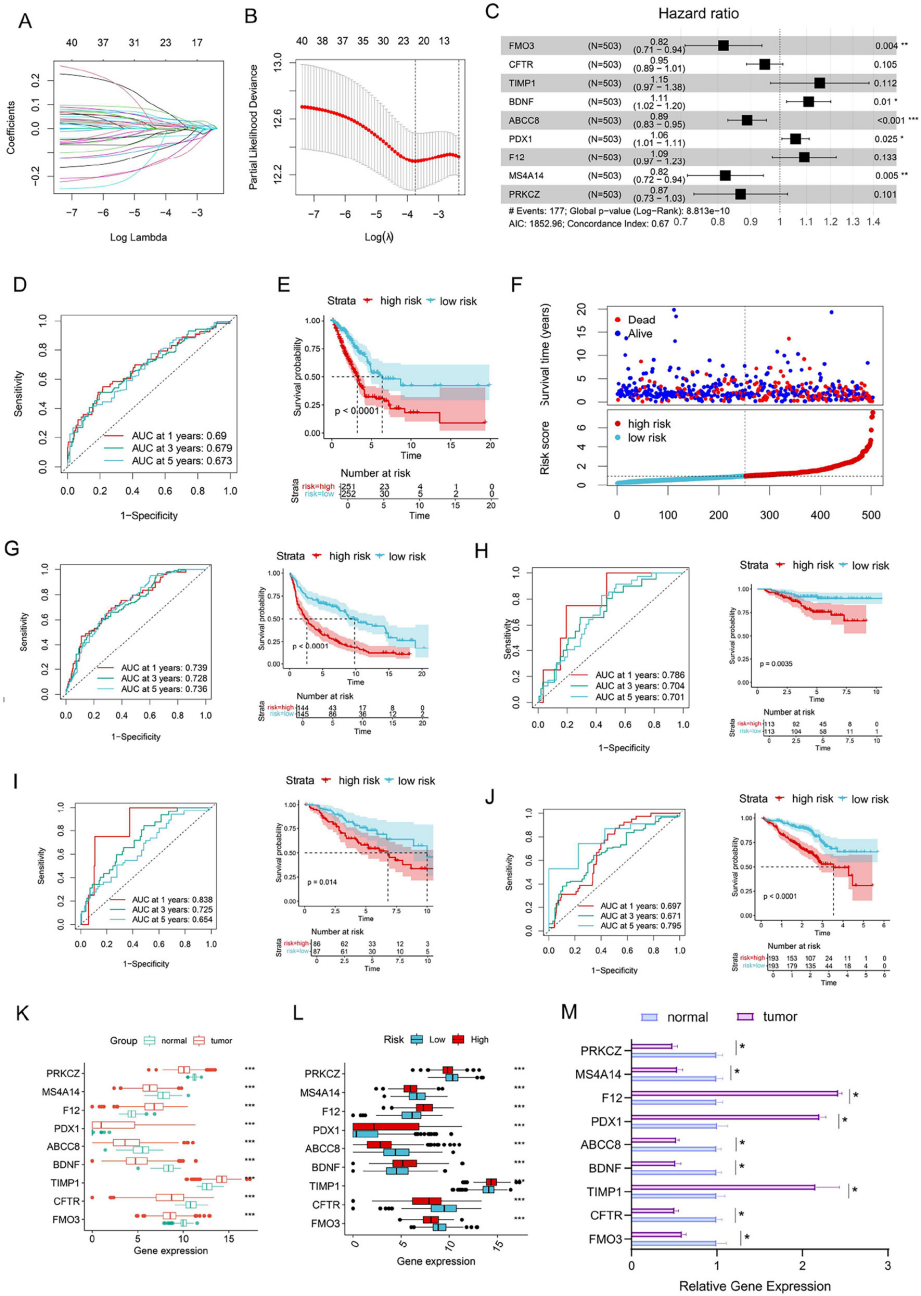
Prognostic model development and validation. (A) LASSO coefficient profiles. (B) λ-selection cross-validation curve. (C) Multivariate Cox regression identifying nine final signature genes. (D) ROC curves for survival prediction in training cohort. (E) Kaplan–Meier survival curves, (F) risk score distribution, and survival status stratified by risk score for the training cohort. Validation cohorts GSE30219 (G), GSE31210 (H), GSE42127 (I), and GSE72094 (J) ROC and Kaplan-Meier survival curves. (K) Signature genes: Normal vs. tumor expression. (L) Signature genes: High-risk vs. low-risk expression. (M) qRT-PCR analysis of nine signature genes expression in BEAS-2B and A549 cells. ns: not significant, *p<0.05, **p<0.01, ***p<0.001.

To prevent overfitting, LASSO regression analysis was applied to the 41 prognosis-related genes, resulting in the selection of 23 candidate genes (Figure 4A and B; Supplementary Table 4). Multivariate Cox regression analysis further refined the candidate gene set, ultimately selecting nine signature genes to construct the prognostic model (Figure 4C, Supplementary Table 5). Risk scores computed for clinical samples; training cohort ROC showed 1-/3-/5-year AUCs: 0.69, 0.679, 0.673 (Figure 4D). Kaplan-Meier analysis confirmed significantly worse survival outcomes in the high-risk group (Figure 4E), while risk score distribution and survival status plots further validated the model’s prognostic capacity (Figure 4F). The calibration curves further demonstrated that the model’s predicted probabilities were in robust agreement with the actual survival probabilities (Supplementary Figure 2, Supplementary Table 6). In the four validation cohorts (GSE30219, GSE31210, GSE42127, and GSE72094), the ROC analysis for 1-year, 3-year, and 5-year survival predictions yielded AUC values all exceeding 0.65, and survival analysis similarly indicated poorer outcomes in the high-risk group, with corresponding risk and survival distribution plots (Figure 4G–J). Gene expression analysis revealed significantly higher expression levels of *F12*, *PDX1*, and *TIMP1* in the tumor group compared to normal group (Figure 4K). *F12*, *PDX1*, and *TIMP1* showed significantly elevated expression in high-risk patients (Figure 4L). To confirm the expression patterns of the five biomarkers, qRT-PCR was performed. The results show that the expression of the nine genes is exactly consistent with data mining (Figure 4M). Kaplan-Meier curves per gene confirmed significant survival associations with signature genes’ expression dichotomy (Supplementary Figure 3).

### Enrichment analysis of high/low-risk groups

**Figure 5:**
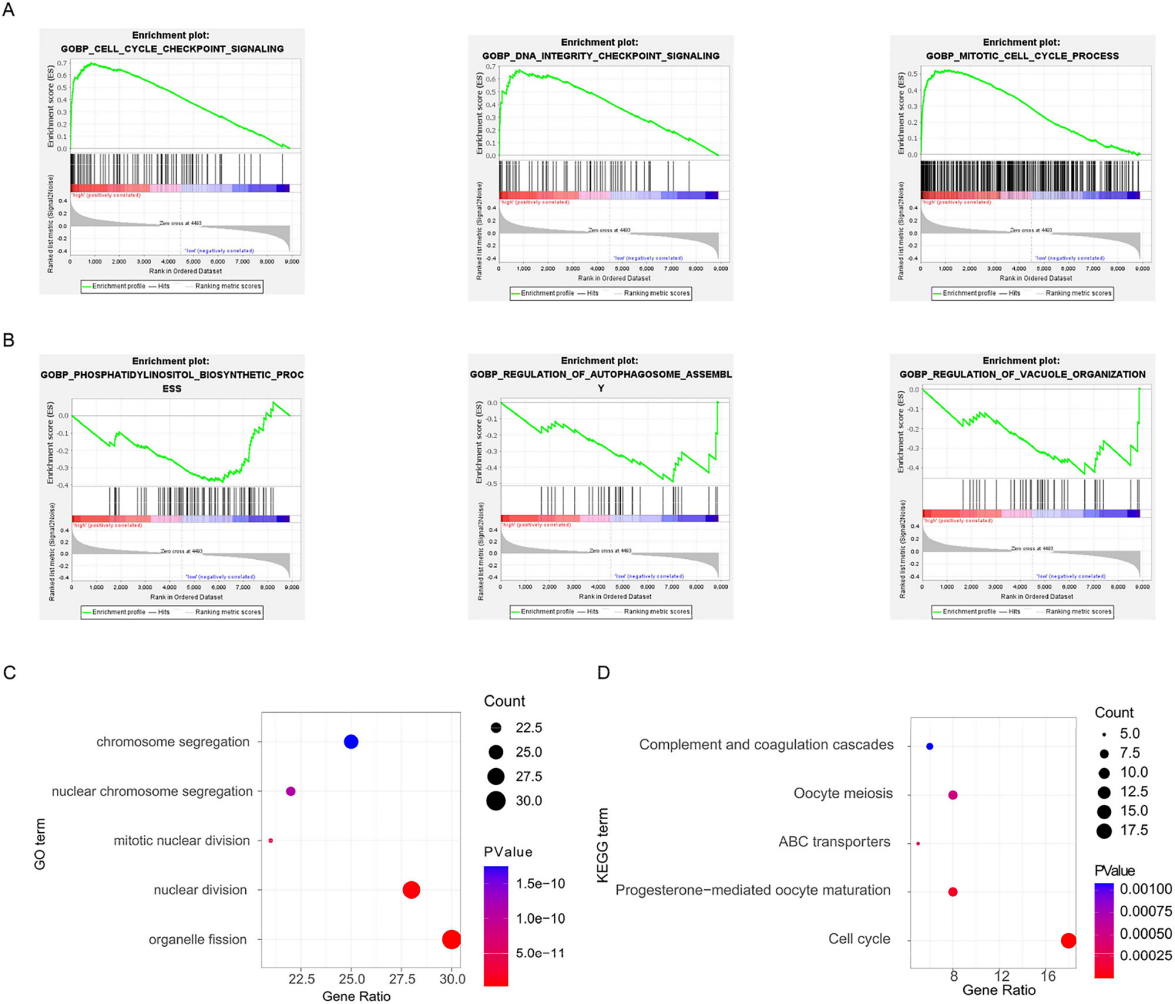
Enrichment analysis between high- and low-risk groups. (A) GSEA: High-risk. (B) GSEA: Low-risk. (C) DEG enrichment: GO. (D) DEG enrichment: KEGG.

GSEA revealed high-risk enrichment in cell cycle checkpoint signaling and DNA integrity checkpoint signaling, while low-risk group including phosphatidylinositol biosynthetic proc and regulation of autophagosome assembly (Figure 5A and B). DEGs analysis using the Wilcoxon test revealed significant enrichment of genes involved in organelle fission, including GO terms related to nuclear division and the cell cycle KEGG pathway (Figure 5C and D).

### Independent prognostic validation and clinical subgroup analysis of the LUAD risk model

**Figure 6:**
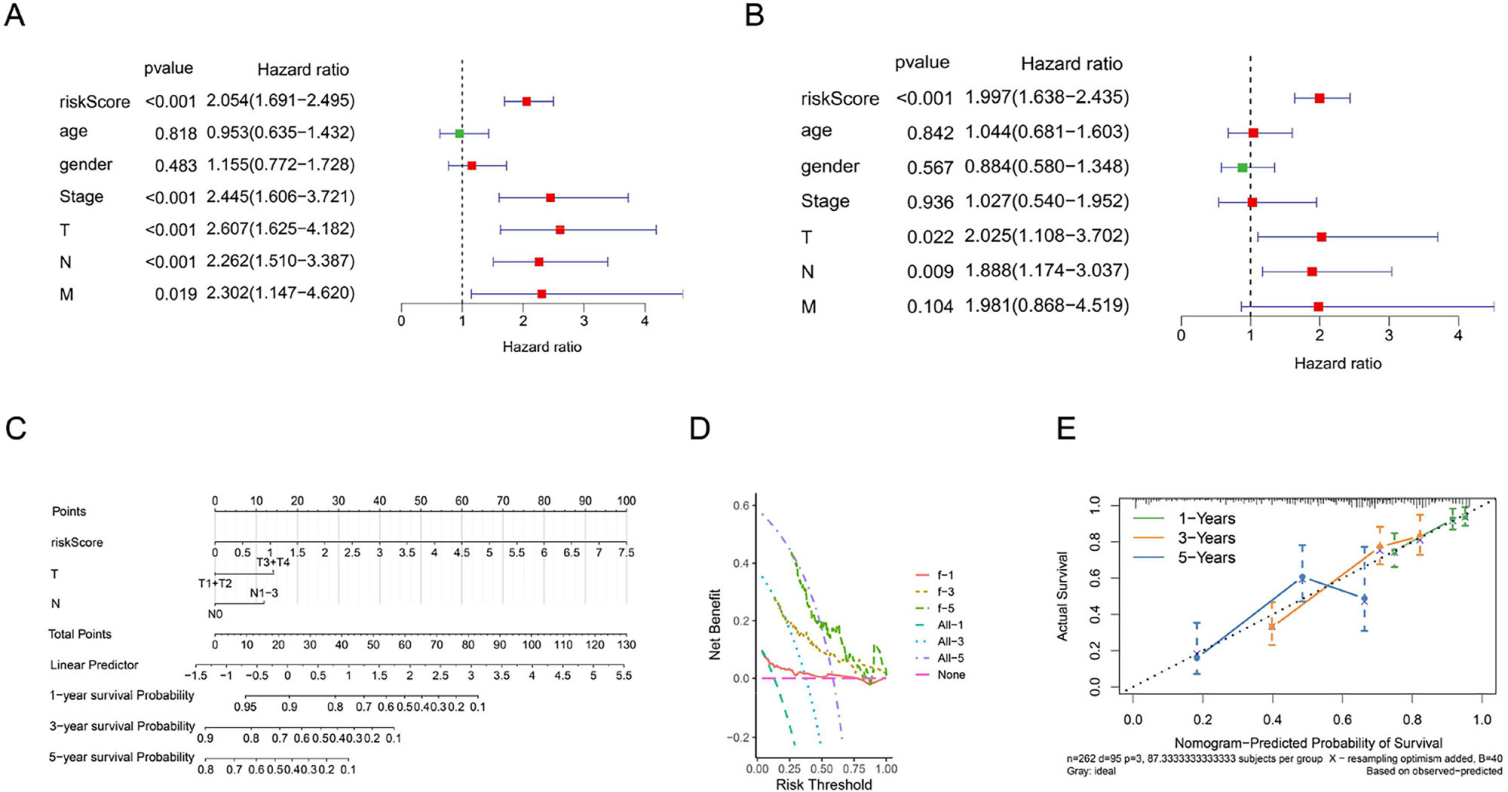
Nomogram survival model: Development and validation. (A) Univariate Cox: Clinicopathologic factors and risk score. (B) Multivariate Cox: Clinicopathologic factors and risk score. (C) Nomogram predicting LUAD prognosis. (D) DCA for 1-/3-/5-year survival. (E) Calibration curves: 1-/3-/5-year survival.

Univariate Cox regression analysis of the LUAD risk model demonstrated significant associations between survival outcomes and multiple variables, including the risk Score, stage, and TNM stage (Figure 6A). Multivariate regression analysis demonstrated that survival outcomes remained significantly associated only with the risk score, T stage, and N stage (Figure 6B). A nomogram incorporating clinical parameters was developed to predict patient survival probability (Figure 6C). DCA confirmed the robust clinical utility of the nomogram across all timepoints (Figure 6D), while calibration curves demonstrated close agreement between predicted and observed survival outcomes (Figure 6E). Clinical characteristics analysis (age, gender, TNM stage) revealed significant risk score differences in: N0 vs. N1-3, T1-2 vs. T3-4, Stage I + II vs. III + IV (Supplementary Figure 4A). Stratified Kaplan–Meier analyses consistently showed reduced overall survival in high-risk subgroups for: age (≤65 vs. >65), stage (I + II vs. III + IV), T (T1-2 vs. T3-4), N (N0 vs. N1-3), M (M0 vs. M1) (Supplementary Figure 4B).

### Risk-stratified immune characterization

**Figure 7:**
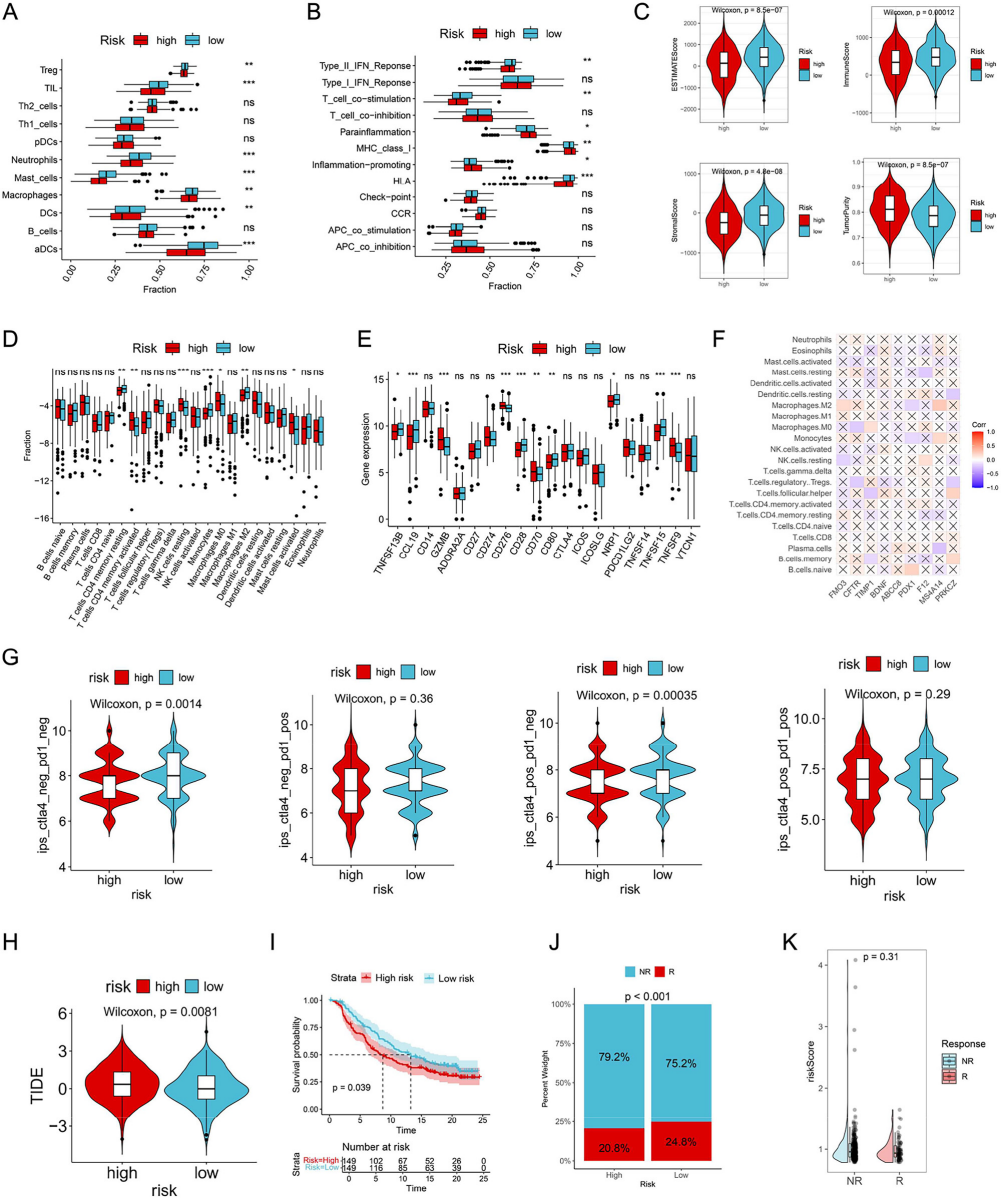
Immune microenvironment characterization. (A) ssGSEA: immune cells. (B) Immune function scores (high- vs. low-risk). (C) ESTIMATE: Microenvironment components. (D) CIBERSORT: Immune fractions (high- vs. low-risk). (E) Immune checkpoint expression (high- vs. low-risk). (F) Signature genes vs. immune infiltration heatmap. (G) IPS scores (high- vs. low-risk). (H) TIDE scores (high- vs. low-risk). (I) IMvigor210 survival (high- vs. low-risk). (J) Immunotherapy response rate (high- vs. low-risk). (K) Risk scores: R vs. NR groups. ns: not significant, *p<0.05, **p<0.01, ***p<0.001.

Based on ssGSEA, the low-risk group exhibited significantly higher levels of Tregs, TILs, macrophages, DCs, neutrophils, aDCs, mast cells, and immune function scores compared to the high-risk group (Figure 7A and B). ESTIMATE analysis revealed significantly higher Immune, Stromal, and ESTIMATE Scores in the low-risk group, whereas the high-risk group exhibited increased Tumor Purity (Figure 7C). CIBERSORT analysis confirmed significantly elevated infiltration of T cells CD4 memory activated, NK cells resting, Macrophages M0, and Mast cells activated in the high-risk group, whereas the low-risk group showed increased infiltration of T cells CD4 memory resting, Monocytes, and Macrophages M2 (Figure 7D). Immune checkpoint gene expression differed significantly between high- and low-risk groups (Figure 7E). The high-risk group demonstrated significantly elevated expression of GZMB, CD276, CD70, and TNFSF9, whereas the low-risk group exhibited markedly higher expression levels of TNFSF13B, CCL19, CD28, CD80, NRP1, and TNFSF15. A correlation heatmap illustrated relationships between immune infiltration and signature gene expression (Figure 7F). The low-risk group showed significantly elevated IPS scores vs. the high-risk group (Figure 7G). TIDE scores differed between risk groups (Figure 7H), and IMvigor210 cohort analysis of PD-L1 blockade response showed significantly better survival in low-risk vs. high-risk patients (Figure 7I). SD (stable disease)/PD (progressive disease) were defined as NR (non-response), while CR (complete response)/PR (partial response) were classified as R (response). The low-risk group showed higher response rates than high-risk (Figure 7J), and R patients had lower risk scores vs. NR patients (Figure 7K).

### Genomic, pharmacogenomic analyses and construction of regulatory networks

**Figure 8:**
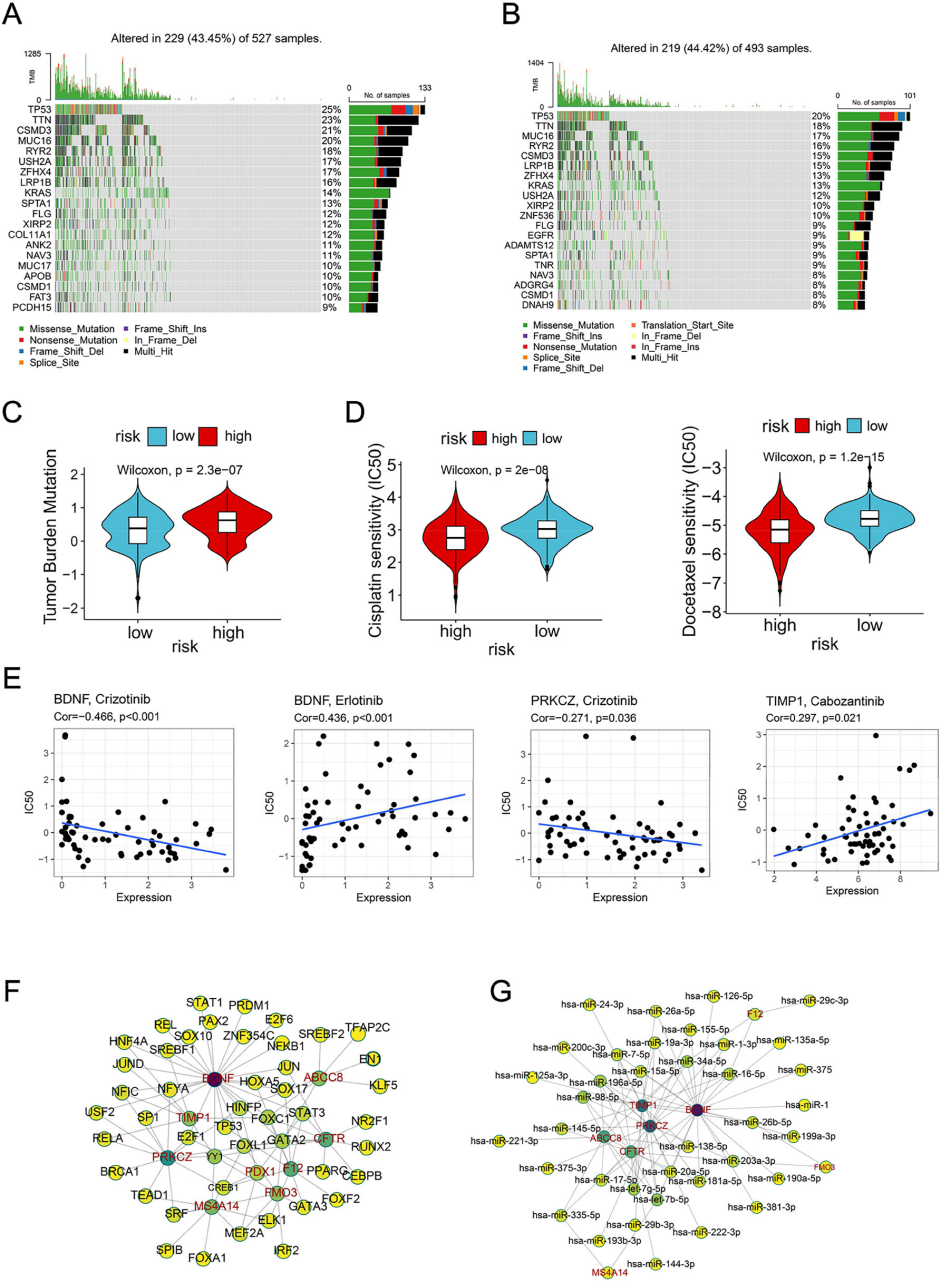
Tumor mutation and drug sensitivity analysis. (A) Top 20 mutated genes (high-risk). (B) Top 20 mutated genes (low-risk). (C) TBM scores: High- vs. low-risk. (D) Drug sensitivity: Cisplatin/docetaxel (high- vs. low-risk). (E) Drug-gene correlations (CellMiner). (F) TF-gene interaction network. (G) miRNA network of signature genes. The higher the node connectivity, the darker the color of the point. Prognostic genes are highlighted in red font.

TMB was calculated using TCGA mutation data across risk groups. Waterfall plots of the top 20 mutated genes revealed high mutation rates in TP53, TTN, and MUC16 in both groups (Figure 8A and B). Comparative analysis demonstrated significantly higher TMB in the high-risk group compared to the low-risk group (Figure 8C). Chemotherapy sensitivity analysis showed lower IC50 values for Cisplatin and Docetaxel in high-risk patients, indicating enhanced drug sensitivity vs. low-risk group (Figure 8D). The MTT cytotoxicity assay results demonstrated that both Cisplatin and Docetaxel exhibited concentration dependent enhanced inhibitory effects on the proliferation of A549 and HCC827 cell lines. A549 cells showed higher sensitivity to both drugs, with a significantly lower IC50 (1.034 μg/mL) for Cisplatin compared to HCC827 cells (6.408 μg/mL, Supplementary Figure 5A), and a markedly lower IC50 (62.31 μm) for Docetaxel (264.3 mm, Supplementary Figure 5B). CellMiner analysis revealed: BDNF negatively correlated with Crizotinib (cor=−0.466) but positively with Erlotinib (cor=0.436); PRKCZ negatively correlated with Crizotinib (cor=−0.271); TIMP1 positively correlated with Cabozantinib (cor=0.297) (Figure 8E, Supplementary Table 2). In the TF network, several transcription factors, including *GATA2*, *STAT3*, and *FOXC1*, are identified as key regulators interacting with multiple prognostic genes, suggesting their central role in transcriptional regulation (Figure 8F). In the miRNA network, several miRNAs such as hsa-miR-98-5p, hsa-miR-196a-5p, and hsa-let-7b-5p demonstrated extensive interactions with the prognostic genes, indicating their potential post-transcriptional regulatory influence (Figure 8G).

## Discussion

In the present study, we found that a novel 9-gene signature based on taurine metabolism effectively serves as a robust prognostic tool for LUAD, confirming our hypothesis. We demonstrated that this risk stratification model not only accurately predicts patient survival outcomes across multiple independent cohorts but also reveals critical heterogeneity in the tumor immune microenvironment. Specifically, our findings indicate that high-risk patients exhibit an immunosuppressive phenotype with genomic instability, whereas low-risk patients are characterized by enhanced immune activity and a more favorable response to immunotherapy.

This nine signature gene delineates the central role of taurine in LUAD pathogenesis. *CFTR* may serve as a prognostic marker for certain specific types of cancer. In patients with breast cancer and colon cancer, low expression of *CFTR* predicts a poor prognosis [19], 20]. In our study, low *CFTR* expression predicted poor prognosis. *FMO3* is expressed in the liver, participates in the metabolism of drugs and xenobiotics, and is involved in cancer through influencing oxidative stress [21]. *TIMP1* has a dual role in cancer: on the one hand, it exerts tumor-suppressive effects by inhibiting matrix metalloproteinases [22]; on the other hand, it can support tumor progression by promoting cell proliferation, inhibiting apoptosis, and other mechanisms [23]. BDNF activates intracellular signaling cascades that support cancer cell growth, survival, proliferation, and migration, thereby contributing to tumor advancement [24]. *ABCC8* encodes the sulfonylurea receptor, which is part of the K-ATP channel [25]. *ABCC8* mRNA independently predicts glioma prognosis and chemosensitivity [26]. *PDX1* regulates pancreatic embryogenesis and suppresses metastasis in pancreatic ductal adenocarcinoma [27]. *F12* is a coagulation factor involved in the intrinsic coagulation pathway [28]. *F12* upregulation in papillary thyroid carcinoma associates with metastasis and poor prognosis [29]. *MS4A14* is generally involved in various biological processes such as cell signaling, cell adhesion, and immune responses [30]. *PRKCZ* is a protein-coding gene that promotes the occurrence of LUAD by sponging miR-766-5p to regulate *MAPK1* [31]. These differential expression patterns collectively establish molecular foundations for LUAD malignancy.

In addition, the four genes F12, PDX1, TIMP1, and BDNF were significantly upregulated in the high-risk group and may directly or indirectly participate in the imbalance of taurine metabolism. The highly expressed F12 and TIMP1 may indirectly deplete or inhibit the antioxidant and metabolic regulatory functions of taurine by inducing pro-inflammatory or pro-proliferative signals, thereby exacerbating malignant proliferation in the high-risk group [32], 33]. As a component of ion channels, ABCC8 may regulate the transport of ions and metabolites across the cell membrane, influencing the transmembrane transport or intracellular concentration of taurine, thereby affecting apoptosis and metabolic homeostasis [34]. CFTR may be involved in the intracellular homeostasis and transport of taurine or its derivatives. The low expression of CFTR limits the protective functions of taurine, leading to an exacerbation of malignant phenotypes [35]. It is noteworthy that the taurine metabolic pathway may be constrained by a broader cellular regulatory network. Studies have indicated that the circadian rhythm regulated by the PER gene family significantly influences cellular functions, metabolic activities, and tumor development [36]. In addition, local environmental factors in the lungs, such as the pulmonary microbiome, also exert profound influences on the immune microenvironment and the development of pulmonary diseases [37]. Future research could explore whether genes associated with taurine metabolism act as downstream targets of circadian rhythm regulation or whether synergistic interactions exist between them. Furthermore, integrating the taurine metabolism risk model with patients’ lung microbiome data could help construct a more comprehensive prognostic and therapeutic response prediction model.

Enrichment analysis showed significant activation of cell cycle/DNA integrity checkpoint signaling in high-risk group. These pathways are directly associated with uncontrolled proliferation and genomic instability in tumor cells, serving as key drivers of cancer progression [38], [39], [40]. Cell cycle checkpoints and DNA integrity checkpoint pathways play crucial roles in cancer: As core guardians of genomic stability, they maintain cellular homeostasis by halting cell cycle progression to allow for DNA damage repair [41], [42], [43]. Notably, taurine can reduce oxidative DNA damage by scavenging ROS, thereby modulating disease progression [44], 45]. Therefore, we propose that the defect in taurine metabolism in the high-risk group leads to a reduction in its antioxidant capacity, resulting in persistently elevated levels of intracellular ROS [46]. High levels of ROS cause sustained DNA damage stress [47]. To cope with this endogenous replicative stress, tumor cells are compelled to compensatorily upregulate cell cycle checkpoints and DNA damage repair pathways to maintain relative genomic stability and avoid apoptosis due to excessive DNA damage [48]. Conversely, phosphatidylinositol biosynthesis and autophagosome assembly regulation pathways were enriched in the low-risk group. This suggests that the regulation of autophagy and lipid metabolism homeostasis may play a protective role in suppressing tumor development [49], 50]. Autophagy is a crucial process for maintaining intracellular homeostasis [51]. In the low-risk group, normal taurine metabolic function can form a synergistic protective axis with autophagy. By scavenging ROS, taurine reduces the production of misfolded proteins and damaged organelles, thereby alleviating the burden on the autophagy system and enabling it to perform homeostatic maintenance functions more efficiently [52]. Autophagy, particularly mitophagy, promptly clears dysfunctional mitochondria, preventing them from generating excessive ROS [53]. Together, they form a positive feedback loop that collectively safeguards cellular energy and redox homeostasis.

Immunotherapy is a current hotspot in oncology research, having achieved a series of breakthrough advances in recent years [54]. For instance, PD-1/PD-L1 inhibitors, as representative immune checkpoint inhibitors, have provided new options for cancer treatment and significantly improved the prognosis for a subset of patients [55]. Particularly for patients with high tumor mutational burden (TMB) or high PD-L1 expression, immunotherapy can yield favorable treatment outcomes [56]. Immune microenvironment differs significantly between risk groups, influencing PD-L1 blockade response and prognosis [57], 58]. The risk model constructed in this study, based on taurine metabolism, can stratify patients into subgroups with distinct tumor immune microenvironments, providing crucial reference value for immunotherapy stratification in LUAD. The low-risk group exhibits an “immune-activated microenvironment,” accompanied by higher immune function scores. This microenvironment suggests the activation of anti-tumor immune responses: resting CD4+ memory T cells serve as an immune memory reservoir, providing a foundation for long-term immune surveillance [59], 60]; while neutrophils may directly kill tumor cells through antibody-dependent cellular cytotoxicity [61], 62]. In the low-risk group, taurine scavenges ROS, thereby reducing the production of misfolded proteins and damaged organelles. This alleviates the burden on the autophagy system, allowing it to maintain cellular homeostasis more efficiently. The central role of taurine as an anti-tumor agent in LUAD has been supported by previous experimental work [12]. Conversely, the high-risk group presents an “immune-desert microenvironment,” [63] characterized by high tumor purity and low immune/stromal scores. This group is enriched with M0 macrophages, which possess strong immunosuppressive effects: M0 macrophages readily polarize into M2-type tumor-associated macrophages, suppressing T cell function and inhibiting immune responses [64], 65]. This microenvironment leads to insufficient T cell infiltration, manifested as low IPS scores and high TIDE scores, indicating high immune escape potential. Consequently, the high-risk group shows a lower response rate to PD-L1 therapy and poorer survival prognosis in the IMvigor210 cohort. In the high-risk group, the accelerated cell cycle and hyperactive DNA repair pathways may drive tumor cells to preferentially respond to stress through proliferation rather than antigen presentation, consequently leading to diminished tumor antigen presentation. In addition, studies have shown that taurine can suppress M1 macrophage polarization by regulating mitophagy and glycolysis, which provides a plausible mechanistic background for the macrophage states (such as the enrichment of M0 macrophages) we observed in high-risk patients [14].

The high-risk group showed lower IC50 than low-risk, suggesting enhanced sensitivity to both drugs. This suggests that the risk model can guide the stratification of treatment strategies. These findings provide a novel strategic framework for clinical stratification: high-risk group may derive significant benefit from conventional chemotherapeutic regimens, whereas low-risk group warrant consideration for combinatorial immunotherapy approaches. These gene-drug correlations can serve as biomarkers to help predict tumor patients’ responses to specific drugs, enabling precision medicine and optimizing treatment strategies to improve therapeutic efficacy while reducing unnecessary side effects.

*STAT3*, *GATA2*, and *FOXC1*, identified as hub transcription factors regulating multiple signature genes, collectively shape tumor microenvironment heterogeneity through coordinated regulation of immune- and development-related pathways. *STAT3*, as a convergence point for numerous oncogenic signaling pathways, plays a crucial role in modulating anti-tumor immune responses and has been extensively studied [66], 67]. *GATA2*, a core transcription factor that has also been extensively studied, suppresses interferon (IFN)-β-mediated anti-tumor immunity in patients with castration-resistant prostate cancer (CRPC), thereby promoting the progression of CRPC [68]. FOXC1, frequently overexpressed across multiple cancer types, is consistently associated with unfavorable clinical outcomes [69].

Although the constructed TMRGs prognostic model holds significant clinical value, it still has certain limitations requiring improvement. This study relies exclusively on computational analyses of retrospective datasets (primarily from TCGA and GEO) and lacks *in vitro* or *in vivo* functional experiments (such as molecular interference or functional assays) to validate the specific mechanistic roles of the nine-gene signature or TMRGs in LUAD. The dependence on retrospective data may introduce sample selection bias, and the diverse clinical treatments received by these patient populations (e.g., chemotherapy, radiotherapy, etc.) and their impact on prognosis were not subjected to detailed stratified discussion in the analysis. Furthermore, the absence of subgroup analyses based on more specific clinical treatment types limits the model’s precision in predicting outcomes in particular clinical contexts. Given these limitations of the computational analyses, future research should prioritize functional validation of these genes and pathways, and explore the integration of technologies such as single-cell transcriptomics and spatial metabolomics to comprehensively elucidate the mechanistic roles of TMRGs and facilitate their clinical translation. Future research will prioritize *in vitro* and *in vivo* experimental validation, including molecular interference targeting nine core characteristic genes and confirming their role in the malignant phenotype of LUAD through functional assays such as cell proliferation, apoptosis, migration, and invasion experiments. Additionally, further validation using prospective, large-sample clinical cohorts is necessary to reduce the bias inherent in retrospective data.

## Conclusions

In summary, our study elucidates the critical involvement of taurine metabolism-related genes in LUAD, providing novel insights into the molecular heterogeneity, immune landscape, and prognostic stratification of patients. The identified gene signature and regulatory networks offer promising avenues for therapeutic intervention and personalized medicine. Future studies should focus on functional validation of these genes and pathways, as well as explore the therapeutic potential of taurine metabolism in LUAD treatment.

## Highlights

– A taurine metabolism-based risk model comprising nine genes has been established to predict the survival rate and immune response in lung adenocarcinoma.
– High-risk and low-risk groups exhibit differential pathway patterns: the high-risk group manifests pro-tumor cell cycle, while the low-risk group demonstrates protective autophagy.
– High-risk group exhibit heightened sensitivity to chemotherapeutic agents, suggesting that this subgroup may derive greater benefit from conventional chemotherapy.

## Supplementary Material

Supplementary Material

Supplementary Material
