# Supplementary material for: Subtyping and risk model construction based on taurine metabolism-related genes for predicting prognosis and immune response in lung adenocarcinoma
Source: Open Med (Wars). 2026 Jul 20;21(1):20261389. doi: 10.1515/med-2026-1389 (PMC13377578; doi:10.1515/med-2026-1389)
Supplement: Supplementary file 1 — Supplementary Material [file j_med-2026-1389_suppl_001.docx]

**Supplementary Figure 1.** **Nonnegative matrix factorization (NMF) rank number screening evaluation (K=2).**

**Supplementary Figure 2.** **Calibration curve of the risk model.**

**Supplementary Figure 3.** **Kaplan-Meier curves.** Kaplan-Meier curves for individual signature genes.

**Supplementary Figure 4.** **Subgroup Analysis of the Clinical Feature-Based Risk Model.** (A) Violin plots of risk score and clinical characteristics vs. survival time. (B) Kaplan-Meier survival curves for clinical subgroups.

**Supplementary Figure 5. MTT assay to detect the viability of A549 and HCC827 cells treated with different concentrations of Cisplatin or Docetaxel for 48 hours.**
